# Genome‐wide association study discovered candidate genes of Verticillium wilt resistance in upland cotton (*Gossypium hirsutum* L.)

**DOI:** 10.1111/pbi.12734

**Published:** 2017-07-08

**Authors:** Tinggang Li, Xuefeng Ma, Nanyang Li, Lei Zhou, Zheng Liu, Huanyong Han, Yuejing Gui, Yuming Bao, Jieyin Chen, Xiaofeng Dai

**Affiliations:** ^1^ Laboratory of Cotton Disease Institute of Food Science and Technology Chinese Academy of Agricultural Sciences Beijing China; ^2^ Xinjiang Academy of Agricultural and Reclamation Science Xinjiang China

**Keywords:** cotton, Verticillium wilt, genome‐wide association, candidate gene, virus‐induced gene silencing (VIGS)

## Abstract

Verticillium wilt (VW), caused by infection by *Verticillium dahliae*, is considered one of the most yield‐limiting diseases in cotton. To examine the genetic architecture of cotton VW resistance, we performed a genome‐wide association study (GWAS) using a panel of 299 accessions and 85 630 single nucleotide polymorphisms (SNPs) detected using the specific‐locus amplified fragment sequencing (SLAF‐seq) approach. Trait–SNP association analysis detected a total of 17 significant SNPs at *P* < 1.17 × 10^–5^ (*P* = 1/85 630, –log_10_ *P* = 4.93); the peaks of SNPs associated with VW resistance on A10 were continuous and common in three environments (RDIG2015, RDIF2015 and RDIF2016). Haplotype block structure analysis predicted 22 candidate genes for VW resistance based on A10_99672586 with a minimum *P*‐value (–log_10_ *P* = 6.21). One of these genes (CG02) was near the significant SNP A10_99672586 (0.26 Mb), located in a 372‐kb haplotype block, and its *Arabidopsis* AT3G25510 homologues contain TIR‐NBS‐LRR domains that may be involved in disease resistance response. Real‐time quantitative PCR and virus‐induced gene silencing (VIGS) analysis showed that CG02 was specific to up‐regulation in the resistant (R) genotype Zhongzhimian2 (ZZM2) and that silenced plants were more susceptible to *V. dahliae*. These results indicate that CG02 is likely the candidate gene for resistance against *V. dahliae* in cotton. The identified locus or gene may serve as a promising target for genetic engineering and selection for improving resistance to VW in cotton.

## Introduction

Verticillium wilt (VW) is caused by the soil‐borne fungus *Verticillium dahliae*, which has a broad host range of over 400 plant species, including economically important cotton, and can survive in soil for many years (Zhang *et al*., 2016a). VW was first reported in Virginia, USA, in 1914 and is found in almost all cotton‐growing areas worldwide. Infected plants typically exhibit symptoms including vascular discoloration, leaf chlorosis, curling or necrosis, followed by defoliation and eventually wilt and plant death as the disease progresses (Bell and Hillocks, 1992; Li *et al*., 2016). VW is a notorious and devastating disease that results in major losses in the yield and quality of cotton (Cai *et al*., 2009; Xu *et al*., 2011); it has been considered one of the most important fungal diseases, inspiring considerable research to develop control measures.

Developing resistant cultivars is an effective and economic approach to controlling wilt disease. Most commercial cultivars are susceptible or only slightly resistant to VW, and cotton breeders mainly rely on sources with partial resistance for cultivar improvement (Wang *et al*., 2008). Thus, to improve VW resistance, cotton breeders must conduct introgression and gene pyramiding from different VW‐resistant sources using marker‐assisted selection (MAS) (Zhao *et al*., 2014). The development and utilization of molecular markers in quantitative trait locus (QTL) mapping provides further information about the genetics of VW resistance in cotton. With the assistance of markers tightly linked to VW resistance, it is possible to transfer resistant genes to develop cultivars with high resistance to VW (Wang *et al*., 2008). To date, more than 100 QTLs conferring VW resistance have been mapped on 22 chromosomes of tetraploid cotton, with the exception of chromosomes 6, 10, 12 and 18, from interspecific populations of *Gossypium hirsutum* × *G. barbadense* (Bolek *et al*., 2005; Du *et al*., 2004; Fang *et al*., 2013; Yang *et al*., 2008; Zhen *et al*., 2006) and from *G. hirsutum* intraspecific populations (Jiang *et al*., 2009; Ning *et al*., 2013; Wang *et al*., 2007; Yang *et al*., 2007) evaluated at different growing stages with different *V. dahliae* isolates. However, genetic loci and molecular markers have not been used effectively in MAS to breed resistant varieties due to limited allelic segregation between the two parents and because fewer recombination events occur in biparental populations.

Genome‐wide association study (GWAS), based on linkage disequilibrium (LD), has emerged as a powerful tool for gene mapping in plants, to take advantage of phenotypic variation and historical recombination in natural populations and overcome the limitations of biparental populations, resulting in higher QTL mapping resolution (Nordborg and Weigel, 2008; Weng *et al*., 2011; Zhu *et al*., 2008). Next‐generation sequencing technologies including genotyping by sequencing (GBS), restriction site‐associated DNA sequencing (RAD‐seq) and specific‐locus amplified fragment sequencing (SLAF‐seq) have been applied for the high‐throughput discovery of high‐quality SNPs for GWAS (Chen *et al*., 2015b; Su *et al*., 2016; Zhao *et al*., 2015b). GWAS has been employed in the breeding of many major crops, such as maize, soya bean, barley, wheat, sorghum and potato, to identify genes responsible for the quantitative variation in complex traits (Breseghello and Sorrells, 2006; Casa *et al*., 2008; Hao *et al*., 2012; Kraakman *et al*., 2006; Malosetti *et al*., 2007; Thornsberry *et al*., 2001). In cotton, GWAS has been performed with respect to yield (Mei *et al*., 2013; Zhang *et al*., 2013) and fibre quality traits (Abdurakhmonov *et al*., 2008; Cai *et al*., 2014; Kantartzi and Stewart, 2008; Zeng *et al*., 2009). A recent GWAS using elite cotton accessions identified molecular markers related to VW resistance, indicating the advantages of GWAS in determining the genetic basis of complex traits in cotton (Zhao *et al*., 2014).

However, few studies have identified the candidate genes underlying VW resistance by GWAS. The tomato *Ve1* and *Ve2* encoding leucine‐rich repeat class of receptor‐like proteins are the only cloned genes that are responsible for VW resistance (Kawchuk *et al*., 2001). Both genes confer resistance when expressed in potato, while only *Ve1* provides resistance in both tomato (Fradin *et al*., 2009) and Arabidopsis (Fradin *et al*., 2011). In cotton, the transcriptomes and proteomes of VW resistance responses have been analysed, and several genes that contribute to the defence response against VW have been reported, including *GbTLP1* (Munis *et al*., 2010), *GbVe1* (Zhang *et al*., 2012), *GbCAD1/GbSSI2* (Gao *et al*., 2013), *GbRLK* (Zhao *et al*., 2015a) and *Hcm1* (Zhang *et al*., 2016b). Expression of *Arabidopsis NPR1* (Parkhi *et al*., 2010), *Hpa1*_*Xoo*_ (Miao *et al*., 2010) and *Nicotiana alata NaD1* (Gaspar *et al*., 2014) in cotton is reported to confer significant resistance to VW.

In this study, we performed a GWAS for VW resistance in the stem of 299 upland cotton accessions genotyped using SLAF‐seq and identified significant associated single nucleotide polymorphism (SNP) loci and potential candidate genes to be verified by virus‐induced gene silencing (VIGS). Our objectives were to supply marker candidates for MAS, identify candidate genes involved in VW resistance and provide insight into the genetic mechanisms of resistance to VW in cotton.

## Results

### Phenotypic characteristics of VW resistance in natural populations

The association panel used in this study consisted of 299 cotton accessions, which were classified into five major groups according to their regional distribution: Yellow River region (YR), Yangtze River region (YZR), Northwest inland region (NW), Liaoning Province (LN) and Foreign group (FG) (Table S1). We evaluated resistance to VW in the 299 accessions in a glasshouse (RDIG2015) and in a field screening nursery in 2015 (RDIF2015) and 2016 (RDIF2016), with three replicates per environment. Extensive phenotypic variation was observed in the disease index (DI), which was calculated and adjusted to a relative disease index (RDI) with a correction factor K. The RDI of all lines ranged from 3.89 to 93.33, with an average of 42.29 in RDIG2015, from 2.72 to 76.67, with an average of 32.10 in RDIF2015, and from 13.06 to 100.00, with an average of 45.31 in RDIF2016 (Table 1). An analysis of variance (ANOVA) of the RDI in three environments revealed significant differences among the genotypes (*F *=* *8.26, *P *<* *0.0001) (Table S2).

**Table 1:** Phenotypic variation in cotton

Environment	Mean	SD	Min	Max	CV(%)
RDIG2015	42.29	21.33	3.89	93.33	50.44
RDIF2015	32.10	17.15	2.72	76.67	53.43
RDIF2016	45.31	19.87	13.06	100.00	43.85

SD, standard deviation; CV(%), coefficient of variation.

### SNP‐based genotyping of the cotton accessions

To finely map the VW resistance genes and investigate beneficial haplotypes in the cotton germplasm, we constructed a haplotype map for the 299 cotton accessions using SLAF‐seq approach. Over 1297 million reads (125 bp each) were generated for the 299 genotypes, encompassing 324.19 Gb of the cotton genomic DNA sequence. Approximately 649 625 high‐quality tags (or SLAFs) were identified from 1297 million paired‐end reads after sequence alignment with the TM‐1 reference genome. The SLAFs used to call SNPs had an average depth of 5.97‐fold per individual among 299 accessions. In total, 884 799 SNPs were initially called for this set of lines; a proportion of these were found to have a minor allele frequency (MAF) of <5%, leaving 85 630 SNPs at MAF ≥ 0.05 and missing rate ≤50% to be used for analysis. The mean genomic distance between SNP tags was approximately 29.2 kb. The data set of 85 630 SNP markers covered all 26 chromosomes. The largest number of SNPs was found on chromosome A08 (8764 SNPs) followed by chromosome A06 (6295 SNPs), and the smallest number of SNPs was found on chromosomes D03 (970 SNPs) and D04 (879 SNPs) (Table S3). The number of SNPs on each chromosome was consistent with the physical length of the chromosome.

### Population structure and linkage disequilibrium

**Figure 1:**
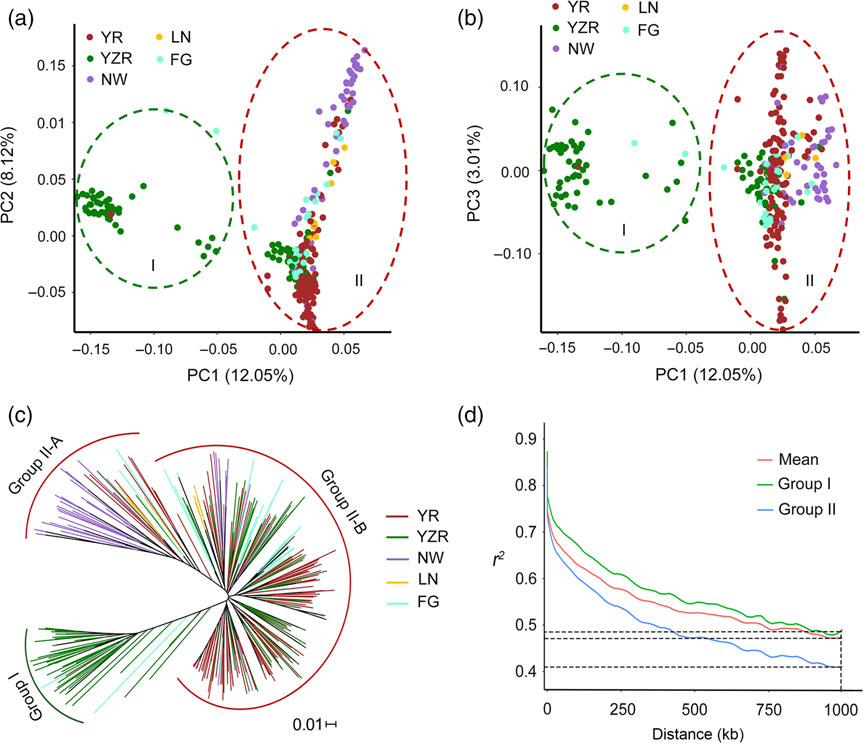
Population structure of the cotton association panel. (a, b) Scatter plots of the first three principal components (PC1, PC2 and PC3) of the panel with colour‐coded groups. Each dot represents an accession; (c) neighbour‐joining tree of 299 cotton accessions in the panel; (d) linkage disequilibrium (LD) decay rate of all cotton accessions and of the two groups (Group I and Group II). The mean LD decay rate was estimated as *r* ^2^, using all pairs of single nucleotide polymorphisms (SNPs) located within 1 Mb of physical distance in 299 cotton germplasm accessions.

A principal component analysis (PCA) was performed using 85 630 SNPs of the 299 accessions in the genome‐wide mapping panel to estimate the influence of population structure on the mapping results. Principal component 1 (PC1) explained 12.05% of the variation in the genotypic data, while PC2 and PC3 explained 8.12% and 3.01% of the variation, respectively. Based on the first three axes of the PCA, the testing accessions were divided into two groups designated Group I (mainly form YZR) and Group II (mainly from YR and NW) (Figure 1a, b). Using the same SNP set, a neighbour‐joining analysis was performed to understand the genetic diversity of our association panel and classified the population into two groups (Figure 1c), which was consistent with the results of PCA. In this study, the first three PCs were added to the compressed mixed linear model (CMLM) for association analysis.

We estimated the LD (indicated by *r* ^2^) decay rate for the entire genome to determine the mapping resolution of the GWAS. The *r* ^2^ was approximately 0.47, where the mean LD decay of our population was 1.0 Mb. Due to variation among different populations, LD decay rates were estimated separately for each of the two groups and revealed that the LD decay distance in Group I was higher than that in Group II (Figure 1d). Given that the marker density was 29.2 kb per SNP, the SNP set was sufficiently dense to capture the genetic variation in the association panel.

### Genome‐wide association analysis

**Figure 2:**
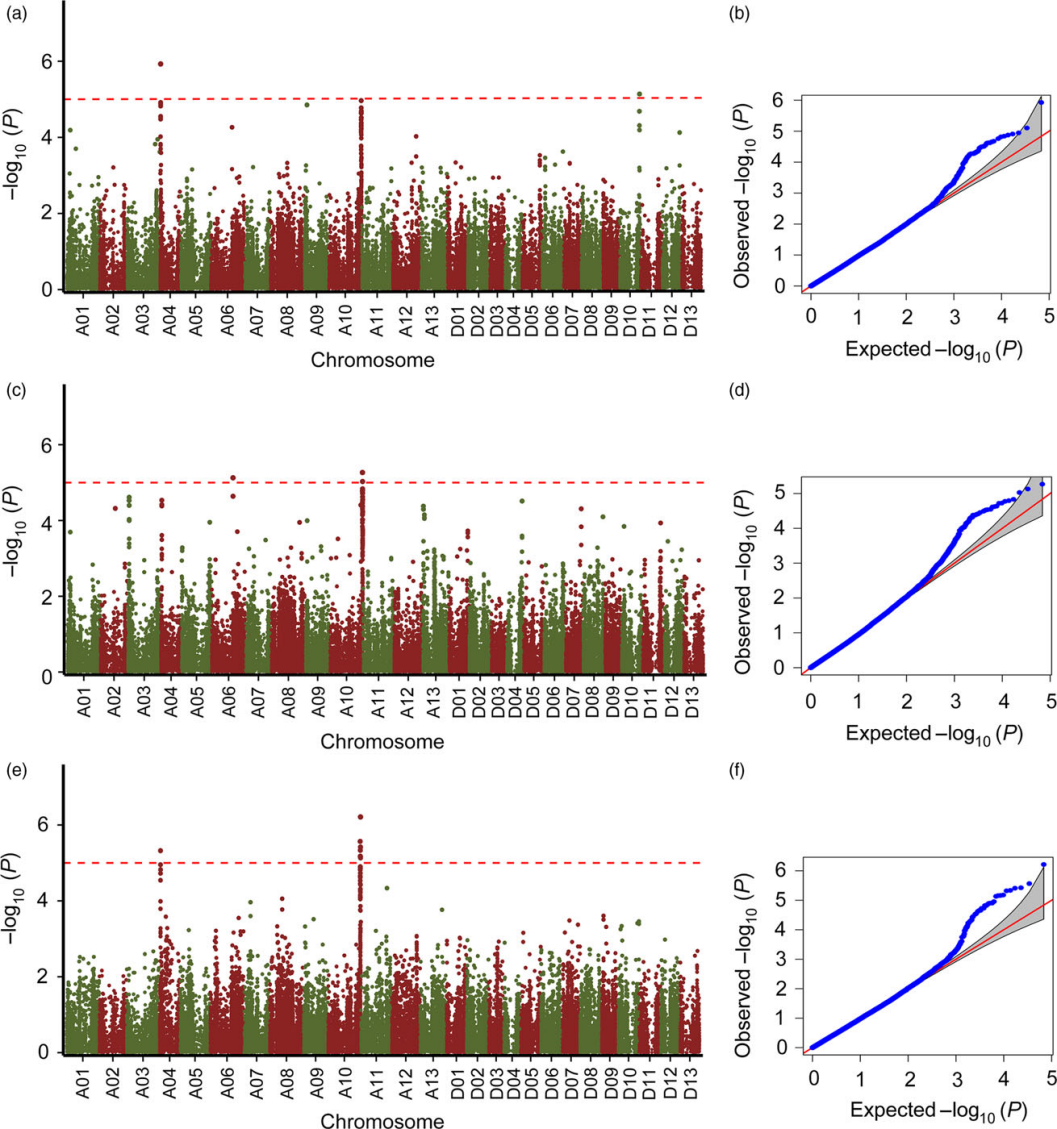
Manhattan and quantile–quantile plots resulting from the GWAS for Verticillium wilt (VW) resistance in cotton. (a, c, e) Manhattan plots for VW resistance in (a) RDIG2015, (c) RDIF2015 and (e) RDIF2016. The *x*‐axis shows the single nucleotide polymorphism (SNPs) along each chromosome; the *y*‐axis is the –log_10_ *P* for the association. The dashed horizontal line indicates the Bonferroni‐adjusted significance threshold (–log_10_ *P* = 4.93); (b, d, f) quantile–quantile plots for VW resistance in (b) RDIG2015, (d) RDIF2015 and (f) RDIF2016.

We performed trait–SNP association analysis for the three environments (RDIG2015, RDIF2015 and RDIF2016) with the CMLM, and which accounts for both population structure and familial relatedness. The quantile–quantile plots showed that the model of the three environments could be used to identify association signals (Figure 2b, d, f). A total of 3, 3 and 11 significant SNPs were detected at *P *<* *1.17 × 10^–5^ (*P *=* *1/85 630, –log_10_ *P* = 4.93) in RDIG2015, RDIF2015 and RDIF2016, respectively (Table 2). Manhattan plots of association analysis showed three peaks (A04, A10 and D10) in RDIG2015, three peaks (A03, A04 and A10) in RDIF2015 and two peaks (A04 and A10) in RDIF2016 (Figure 2a, c, e). Further investigation showed that the A10 peaks were continuous and common to all three environments. Five SNPs (A10_98859056, A10_99071882, A10_99071906, A10_99110423 and A10_99240983) were identified in all three environments among the SNPs at –log_10_ *P* > 4.5 on A10 (Table S4). The first peak, SNP A10_99071906 (*P *=* *1.13 × 10^–5^, –log_10_ *P* = 4.95), was identified in RDIG2015, then A10_98859056 (*P *=* *5.41 × 10^–6^, –log_10_ *P* = 5.27) in RDIF2015 and A10_99672586 (*P *=* *6.14 × 10^–7^, –log_10_ *P *= 6.21) in RDIF2016, which are located in a genomic region with a distance of 0.81 Mb (Table 2).

**Table 2:** SNPs associated with VW resistance

SNP	Position	SNP Allele	MAF	RDIG2015		RDIF2015		RDIF2016	
				−log_10_(*P*)	*R* ^2^ (%)	−log_10_(*P*)	*R* ^2^ (%)	−log_10_(*P*)	*R* ^2^ (%)
A04_2334165	2334165	C/A	0.34	5.92	7.52				
A04_3445154	3445154	A/G	0.07					4.95	4.53
A04_3516941	3516941	A/G	0.07					5.32	4.93
A06_65034874	65034874	A/G	0.25			5.13	6.37		
A10_98700922	98700922	G/A	0.14					5.57	5.19
A10_98859056	98859056	G/A	0.14			5.27	6.57		
A10_99071882	99071882	A/C	0.13					5.18	4.77
A10_99071906	99071906	A/C	0.14	4.95	6.1			5.33	4.94
A10_99110423	99110423	T/C	0.14			5.03	6.22	5.16	4.75
A10_99672524	99672524	T/C	0.17					5.13	4.72
A10_99672553	99672553	A/G	0.17					5.42	5.03
A10_99672585	99672585	G/A	0.17					5.41	5.02
A10_99672586	99672586	C/T	0.17					6.21	5.89
A10_99672605	99672605	G/A	0.17					5.17	4.76
D10_60486237	60486237	C/T	0.24	5.1	6.33				

SNP allele, major and minor alleles; MAF, minor allele frequency; *R* ^2^ (%), percentage of phenotypic variation.

To further confirm these significant SNPs associated with VW, we compared our GWAS results with those of previous linkage studies. Three significant SNPs (A04_2334165, A04_3445154 and A04_3516941) on A04 were mapped to regions where VW resistance QTL (*qVWR‐08‐c4‐1*) had been previously reported (Zhang *et al*., 2015a), adding support to our findings (Figure S1a). Although previously reported VW resistance QTLs were not found on A10, the region of SNPs identified on A10 was enriched with resistance gene analogues (RGAs) that exhibited clusters using bioinformatics methods (Figure S2) (Holub, 2001). Thus, the significant SNPs that we identified on A10 were novel, and we assigned it for further analysis.

### Identification of favourable SNP alleles associated with VW resistance

**Figure 3:**
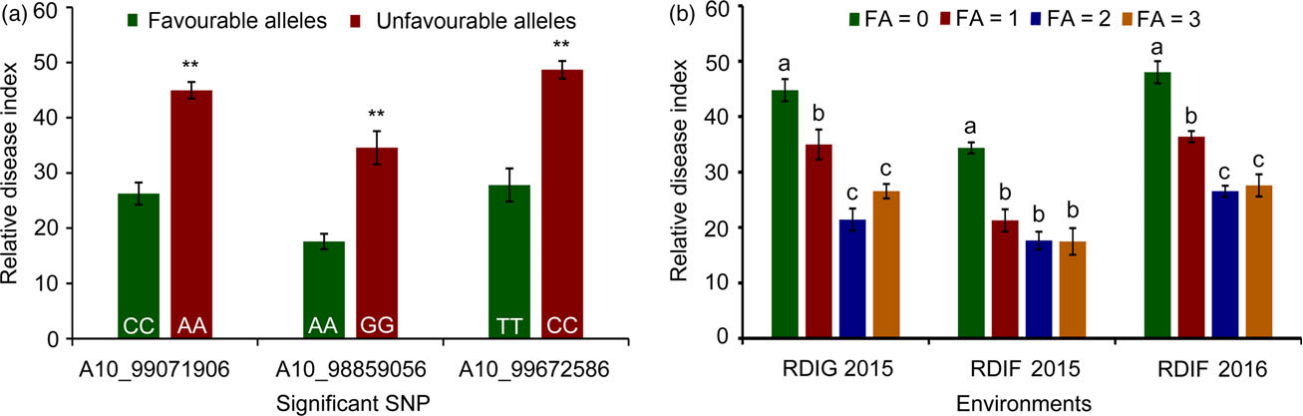
Mining of favourable SNP alleles. (a) Relative disease index of accessions with favourable and unfavourable alleles at the SNPs (A10_99071906, A10_98859056 and A10_99672586) for VW resistance. ** indicates significant difference (*P *<* *0.01); (b) relative disease index of accessions with different numbers of favourable alleles (CC, AA and TT). FA indicates the numbers of favourable alleles. Different letters (a, b, and c) indicate significant difference (*P *<* *0.01).

To understand the effects of allelic variation on VW resistance, we selected three significant SNPs (A10_99672586, A10_98859056 and A10_99071906) on A10 as identified favourable alleles. These SNPs exhibited the minimum *P*‐values (−log_10_ *P* = 6.21, 5.27 and 4.95, respectively), and the *R* ^2^ were 5.89%, 6.57% and 6.1%, respectively, explaining the most phenotypic variation among the significantly associated SNPs. The SNP alleles were C/T, A/G and A/C, respectively. For the first peak SNP (A10_99672586), the average RDI of accessions with favourable alleles (T) was 27.81, which was lower than the average RDI (48.68) of accessions with unfavourable alleles (C) investigated in RDIF2016. The RDI values of accessions with favourable alleles (A) at A10_98859056 (17.58) and (C) at A10_99071906 (26.26) were both lower than the average RDI values for accessions with unfavourable alleles (G) at A10_98859056 (34.57) and (A) at A10_99071906 (44.98) (*P *<* *0.01) (Figure 3a). These results indicate that the phenotypic characteristics of the genotypes with favourable SNP alleles were significantly enhanced compared to those of the genotypes with unfavourable SNP alleles.

The mean RDI values for accessions that contained different numbers of favourable SNP alleles indicate that lower RDI values occurred in the cotton accessions with favourable SNP alleles compared to those either without such alleles or with fewer such alleles. The average RDI values for accessions without favourable alleles were 34.36, 48.01 and 44.78 in RDIF2015, RDIF2016 and RDIG2015, respectively; the average RDI values for accessions with a single favourable allele were 21.28, 36.40 and 34.97, respectively. The average RDI values for accessions with two or three favourable alleles were lower significantly than those with zero or a single favourable allele (*P *<* *0.01). These results indicate that favourable SNP alleles had significant pyramiding effects on VW resistance (Figure 3b).

### Prediction of candidate genes of VW resistance

**Figure 4:**
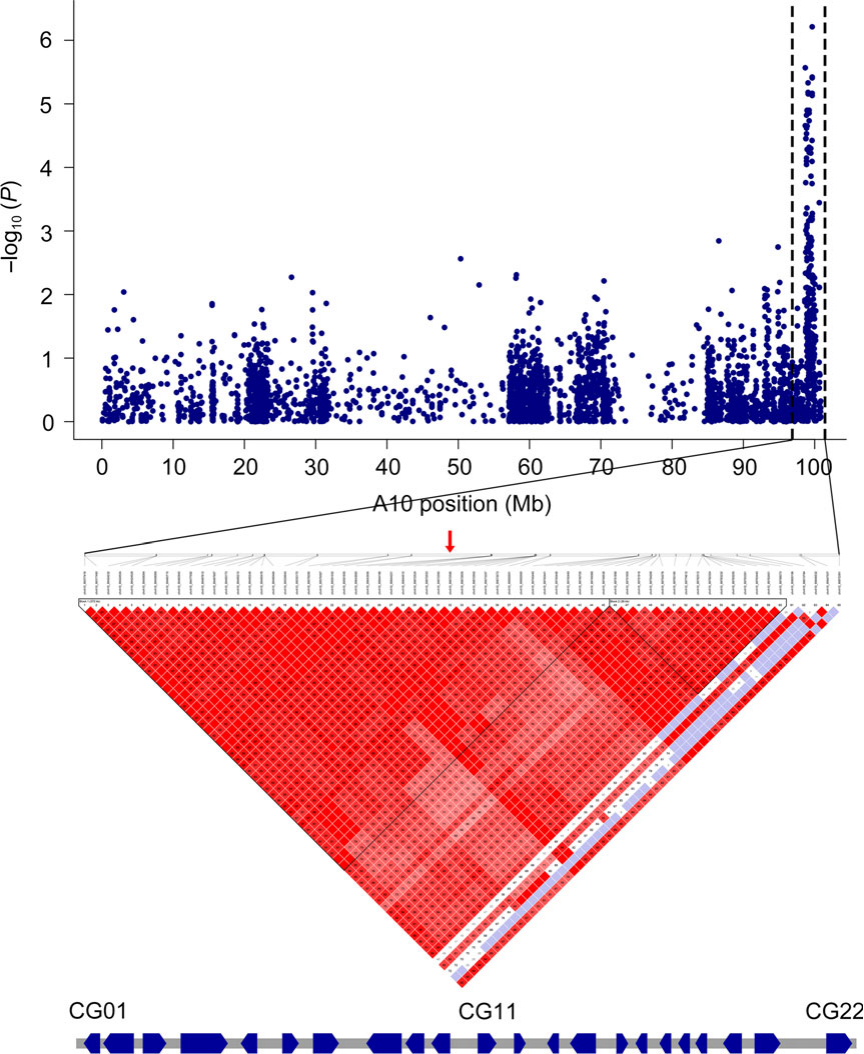
Genomic location of SNP locus associated with VW resistance and haplotype block analysis. Red arrows denote significantly associated SNPs located in the haplotype block. Genes within this region are indicated below.

The first peak SNP (A10_99672586) on A10 exhibited the minimum *P*‐value (–log_10_ *P* = 6.21) in RDIF2016, and may be a major genetic locus responsible for VW resistance in cotton. Thus, we investigated the haplotype block structure based on A10_99672586, within 300 kb on either side, to determine candidate genes. Block structures showed that the first peak SNP (A10_99672586) was involved in a 372‐kb haplotype block encompassed by 45 SNPs (Figure 4). Haplotype block structure analysis for A10 indicated that the candidate gene regions were 99.38–99.75 Mb, and a total of 22 genes were found on A10. A gene ontology analysis showed that three genes were without any definite annotation concerning their biological function and four genes had unpredicted pathways. Four genes were linked to biological pathways involved in plant stress response, including disease resistance, and the other genes were predicted to be involved in transport, translational regulation, transcription regulation and signal transduction, among other processes (Table 3).

**Table 3:** Candidate genes linked genomic region of SNP most highly associated with VW resistance in cotton

Code	Gene	Start	Stop	*Arabidopsis* homologues	Description	GO annotation
CG01	Gh_A10G2075	99409815	99410634		Hypothetical protein	Function unknown
CG02	Gh_A10G2076	99411620	99415320	AT3G25510	Disease resistance protein (TIR‐NBS‐LRR class), putative	Defence response
CG03	Gh_A10G2077	99423054	99425281	AT4G16890	Disease resistance protein (TIR‐NBS‐LRR class), putative	Response to stimulus
CG04	Gh_A10G2078	99443579	99452906	AT3G25510	Disease resistance protein (TIR‐NBS‐LRR class), putative	Response to stimulus
CG05	Gh_A10G2079	99466199	99467209	AT3G09250	Nuclear transport factor 2 (NTF2) family protein	Function unknown
CG06	Gh_A10G2080	99507293	99508656	AT1G69550	Disease resistance protein (TIR‐NBS‐LRR class)	Pathway unpredicted
CG07	Gh_A10G2081	99521849	99524748	AT3G25510	Disease resistance protein (TIR‐NBS‐LRR class), putative	Signal transduction
CG08	Gh_A10G2082	99547079	99552644	AT4G13360	ATP‐dependent caseinolytic (Clp) protease/crotonase family protein	Proteolysis
CG09	Gh_A10G2083	99555764	99557360	AT3G24450	Heavy metal transport/detoxification superfamily protein	Cellular transition metal ion homeostasis
CG10	Gh_A10G2084	99561564	99563596	AT3G24460	Serinc domain containing serine and sphingolipid biosynthesis protein	Pathway unpredicted
CG11	Gh_A10G2085	99601580	99603022	AT2G39980	HXXXD‐type acyl‐transferase family protein	Pathway unpredicted
CG12	Gh_A10G2086	99613022	99614422	AT2G39980	HXXXD‐type acyl‐transferase family protein	Pathway unpredicted
CG13	Gh_A10G2087	99631484	99632864	AT3G11260	WUSCHEL related homeobox 5	Regulation of transcription
CG14	Gh_A10G2088	99640405	99643453	AT2G39990	Eukaryotic translation initiation factor 2	Regulation of translational initiation
CG15	Gh_A10G2089	99656268	99657394	AT4G00430	Plasma membrane intrinsic protein 1;4	Transport
CG16	Gh_A10G2090	99664460	99665584	AT4G00430	Plasma membrane intrinsic protein 1;4	Transport
CG17	Gh_A10G2091	99675191	99676314	AT4G00430	Plasma membrane intrinsic protein 1;4	Transport
CG18	Gh_A10G2092	99682369	99683695	AT2G29670	Tetratricopeptide repeat (TPR)‐like superfamily protein	Pathway unpredicted
CG19	Gh_A10G2093	99688252	99689673	AT2G40000	Orthologue of sugar beet HS1 PRO‐1 2	Defence response
CG20	Gh_A10G2094	99701603	99703792	AT2G40010	Ribosomal protein L10 family protein	Cytoplasmic translation
CG21	Gh_A10G2095	99705983	99708239	AT5G05760	Syntaxin of plants 31	Transport
CG22	Gh_A10G2096	99741994	99744974	AT2G38120	Transmembrane amino acid transporter family protein	Transport

GO annotation of biological process was indicated in the table.

**Figure 5:**
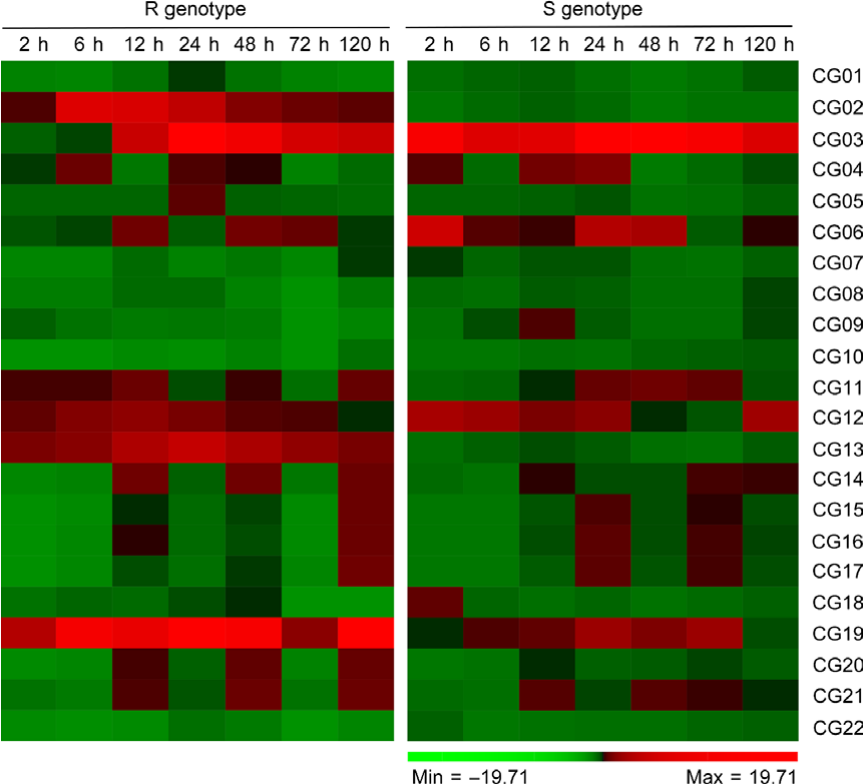
Heat map for expression patterns of the candidate genes at 2, 6, 12, 24, 48, 72 and 120 h postinoculation with *V. dahliae* strain Vd991 in R genotype (Zhongzhimian2) and S genotype (Jimian11). Red represents high expression; green represents low expression.

To investigate which genes were responsible for resistance to VW, we used quantitative real‐time PCR (qRT‐PCR) analysis to determine differential gene expression in candidate genes identified by GWAS analysis. The results showed that five genes (CG02, CG03, CG12, CG13 and CG19) were up‐regulated in the resistant (R) genotype Zhongzhimian2 (ZZM2) and susceptible (S) genotype Jimian11 (JM11) at different times after *V. dahliae* inoculation (Table S5). Two genes (CG02 and CG13) specific to the R genotype were implicated in resistance against *V. dahliae* in cotton (Figure 5). *Arabidopsis* homologues of CG02 and CG13 encode TIR‐NBS‐LRR protein and WOX family transcription factors, respectively, which have been proposed to be involved in disease resistance response and signal transduction.

### Potential functional roles of CG02 in *V. dahliae* resistance

**Figure 6:**
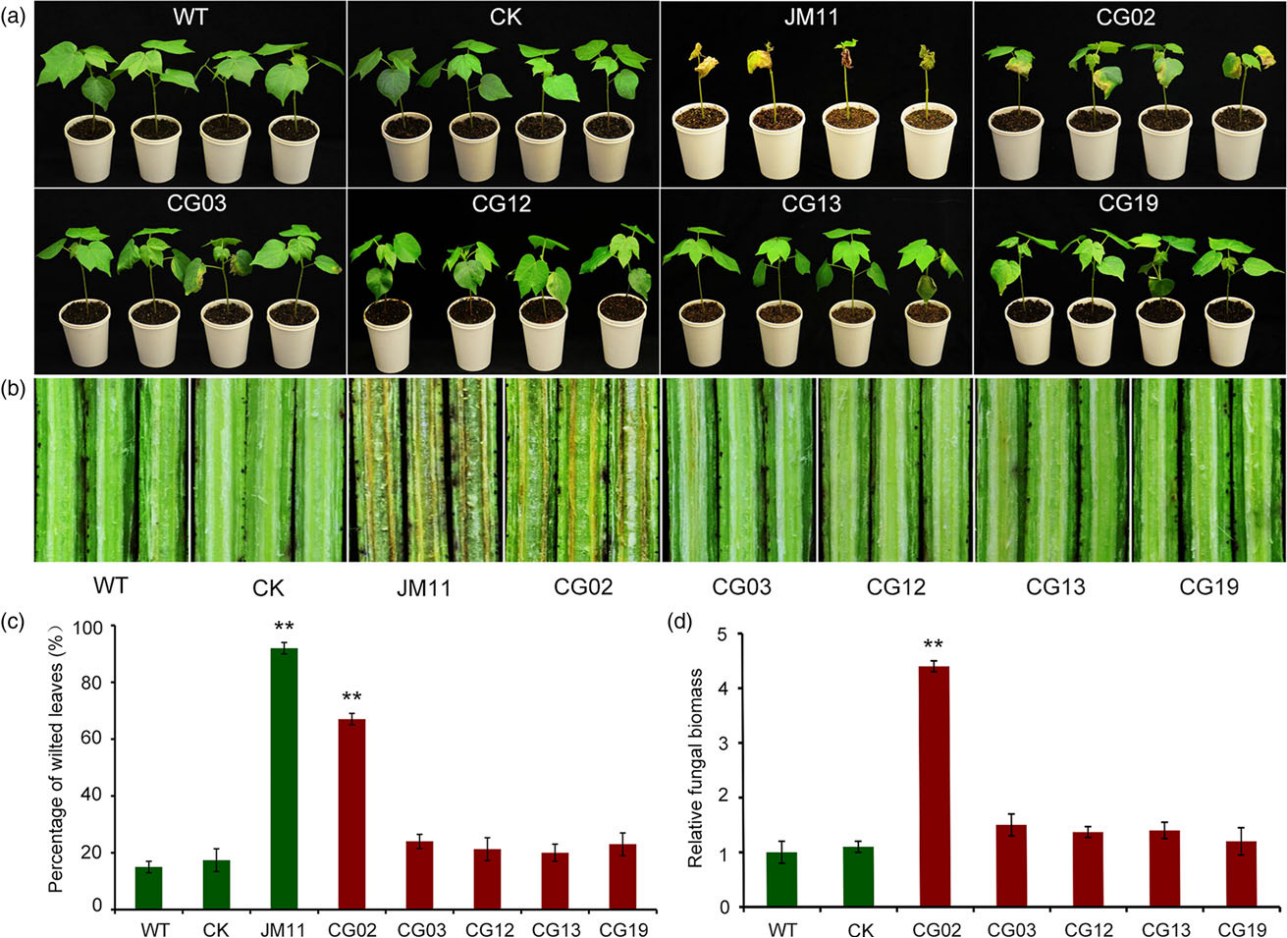
Analysis of five candidate genes implicated in *V. dahliae* resistance as determined by VIGS. (a) Plant phenotypes at 21 day postinoculation; (b) stem symptoms of control and silenced plants inoculated with *V. dahliae* strain Vd991; (c) percentage of diseased leaves after *V. dahliae* inoculation. Experiments were performed using 20 plants per treatment; (d) fungal biomass determined by qRT‐PCR in control and gene‐silenced plants. Experiments were performed using at least nine inoculated plants and the Verticillium elongation factor 1‐α gene was used for equilibration of transcript levels. Error bars were calculated based on three biological replicates using standard deviation. WT and CK represent Zhongzhimian2 (ZZM2) and pTRV2:00, respectively. CG02, CG03, CG12, CG13 and CG19 represent gene‐silenced plants. ** indicates significant difference (*P *<* *0.01).

To further investigate the function of the five R genotype up‐regulated genes in VW resistance, we performed VIGS, constructing recombinant viruses including pTRV2:CG02, pTRV2:CG03, pTRV2:CG12, pTRV2:CG13 and pTRV2:CG19 to silence endogenous genes, with pTRV1 serving as a mock treatment. When plants infiltrated with pTRV2:CLA1 showed bleaching in newly emerged leaves, we used qRT‐PCR to confirm the silencing of the genes, which exhibited lower expression levels in five genes in infiltrated pTRV2:CG02, pTRV2:CG03, pTRV2:CG12, pTRV2:CG13 and pTRV2:CG19 plants than the control (ZZM2, WT and pTRV2:00, CK) (Figure S3). We inoculated these plants by dip infection with conidial suspension (5 × 10^6^ conidia/mL). After 3 weeks, the control plants seldom exhibited leaf wilting; approximately 15% of plants were diseased. Among the gene‐silenced plants, 27% of CG03‐silenced plants, 21% of CG12‐silenced plants, 20% of CG13‐silenced plants and 23% of CG19‐silenced plants exhibited the wilting phenotype when infected with *V. dahliae*, indicating no significant difference from the proportion of control plants. Furthermore, 67% of CG02‐silenced plants were severely infected by *V. dahliae*, similar to the proportion observed in susceptible control plants JM11, with 92% diseased plants; (Figures 6a, c and S4). The vascular wilt symptoms varied; the stems of CG02‐silenced plants turned brown, whereas other gene‐silenced plants showed no wilt symptoms in stems (Figure 6b). Fungal biomass qRT‐PCR analysis demonstrated that CG02‐silenced plants developed significantly higher fungal biomass than control plants, 4.3‐fold higher than that in WT and 3.9‐fold higher than in CK, whereas fungal biomass in CG03‐, CG12‐, CG13‐ and CG19‐silenced plants did not differ significantly from that in control plants (Figure 6d). These results strongly indicate that CG02 is required for *V. dahliae* resistance in cotton.

## Discussion

### GWAS and population structure

VW caused by *V. dahliae* is one of the most destructive diseases in cotton. Understanding the mechanism of host resistance to VW through gene/QTL analysis and breeding resistant cultivars through MAS are thought to be the most practical and effective ways to manage this disease. However, it is difficult to reliably identify VW resistance QTLs for various reasons, including limited biparental allelic separation and the low genome coverage of molecular markers (Fang *et al*., 2013; Zhang *et al*., 2014a, 2015a; Zhou *et al*., 2014). In addition to the small effects of most loci, the limited allelic segregation and recombination of biparental populations in linkage mapping hinder the identification of VW resistance QTLs or genes. Moreover, most previous studies on QTL mapping for VW resistance in cotton have been based on early‐segregating mapping populations that could not be evaluated repeatedly, causing higher experimental errors (Wang *et al*., 2007; Wu *et al*., 2009; Zhen *et al*., 2006). In this study, we used an association population consisting of 299 upland cotton accessions from broader breeding populations for VW resistance QTL detection, which can offer more historical recombination events to overcome the limitations of biparental populations.

We performed PCA to ascertain the divergence of the populations in our panel. The first three principal components were correlated with geographical distribution and defined two groups, although different degrees of introgression were detected within these groups. The accessions of Group I mainly derived from YZR and Group II mainly derived from YR (Group II–A) and NW (Group II–B), which was consistent with the results of phylogenetic analysis (Figure 1c). The accessions from LN and FG were more dispersive than those of the other groups (Figure 1a, b). We suppose that these accessions are exchanged frequently during the cultivation process. We also found that some accessions from Jiangsu and Jiangxi provinces in YZR are more closely related to the YR group and present greater genetic introgression from the YR group (Figure 1a, b; Table S1); this result suggests that the accessions from Jiangsu and Jiangxi might initially derive from YR cotton.

### Screening for VW resistance in cotton

In this study, we evaluated a population of 299 accessions for VW resistance under field screening nursery and glasshouse inoculation conditions. Fields heavily infested with *V. dahliae* can serve as long‐term or permanent natural nurseries for screening and selecting cotton for VW resistance (Bell and Hillocks, 1992); however, it is difficult to maintain a relatively uniform distribution of inocula at high density under field conditions. Seasonally variable environmental factors and weather also affect screening results (Zhang *et al*., 2014a). Because VW is highly sensitive to environmental and developmental factors, in‐field evaluation can be a challenge in achieving reliable VW resistance screening results. Due to the limitations of field evaluation of VW resistance, we performed artificial inoculation in a glasshouse. In glasshouse assays, cotton plants with two true leaves were infected by root inoculation using root‐dipping techniques (Liu *et al*., 2013). A defoliating *V. dahliae* isolate Vd991, abundant in the Yellow River and Yangtze River cotton regions (Zhang *et al*., 2014b), was used for disease infection in our glasshouse assay. A single *V. dahliae* isolate infection could allow accurate and effective identification of resistant accessions under the same criteria. However, biological limitations are associated with using a single isolate, which cannot represent the diversity that would generally be observed in the field, where mixed populations of *V. dahliae* would typically be found (Wei *et al*., 2015). A number of *V. dahliae* isolates may be found in the field; 67 isolates of *V. dahliae* have been identified in pepper fields belonging to different vegetative compatibility groups (Bhat *et al*., 2003). Due to this variability in distribution in field and glasshouse environments, different QTLs should be identified in cotton associated with resistance to *V. dahliae* isolates. Zhao *et al*. (2014) showed that 15 and 32 markers were significantly associated with VW resistance in cotton in a glasshouse and a disease nursery, respectively; five of these markers were significantly associated with VW resistance in both environments. Given this previous result, we performed two assays under field and glasshouse conditions to identify the overlapping QTLs associated with VW resistance in cotton. We found that three peaks on A10 identified in three environments were overlapped (Figure 2) and five SNPs were common at –log_10_ *P* > 4.5 (Table S4).

It is recommended to perform multiple replicated tests to reduce experimental errors in evaluating VW resistance and provide more reliable phenotypic data for genetic studies and QTL mapping (Fang *et al*., 2013; Ning *et al*., 2013). We screened 60 plants with three replicates for each genotype (20 plants per genotype) for VW resistance in the field and 90 plants with three replicates (30 plants per genome) in the glasshouse after inoculation with *V. dahliae* isolate Vd991. This improved screening method for evaluating VW resistance was performed for more efficient and effective QTL mapping (Zhang *et al*., 2014a).

### Associated SNP comparison with known QTLs

We used GWAS to detect SNPs associated with VW resistance in cotton and identified 17 SNPs that had significant association with *V. dahliae* response. To further confirm these loci for VW resistance, a comparison of the GWAS was performed with QTLs identified in previous studies. A total of 85 QTLs for VW resistance containing 139 simple sequence repeat (SSR) markers were selected from 11 QTL mapping reports (Table S6). The physical locations of these SSR primer sequences were mapped to the reference genome sequence (Zhang *et al*., 2015b) via electronic PCR (e‐PCR). A previous report evaluated VW resistance in a backcross inbred line population, and a QTL (*qVWR‐08‐c4‐1*) was detected in the region of 0.97–3.87 Mb on A04 (Zhang *et al*., 2015a). Three significant SNPs (A04_2334165, A04_3445154 and A04_3516941) on A04 as identified by GWAS were positioned between BNL3089 and NAU3469 of the QTL *qVWR‐08‐c4‐1* (Figure S1a). Furthermore, some peak SNPs on A02, A13, D05 and D12 identified in this study overlapped with previous QTLs, although they were lower than the threshold (*P *<* *1.17 × 10^–5^, –log_10_ *P* = 4.93) (Figure S1b). The SNP (A02_45851582) located on the A02 peak in RDIF2015 was positioned between MUSS294 and NAU1072 in QTL *qFDI711‐30‐0.01* (Wu *et al*., 2010). Peak SNPs A13_2361609 identified in RDIG2015 and A13_2361299 in RDIF2015 were positioned between NAU2730 and NAU5110 in *qVWI‐09‐c13‐1* (Zhang *et al*., 2015a). Peak SNP D05_54737235 in RDIG2015 was mapped to the regions of QTLs *qFDI711‐27‐26.01* and *qVV‐D5‐1BC1S2592* (Wu *et al*., 2010; Yang *et al*., 2008). Peak SNP D12_11092664 identified in RDIF2015 was positioned between BNL3867 and BNL1605 in *q7.22‐2* (Wang *et al*., 2008). These results show that the loci for VW resistance identified in this study had considerable overlap with previously reported loci, suggesting that a GWAS with a panel of 299 cotton accessions is suitable for the identification of significant SNPs associated with VW resistance. Although the significant SNPs identified on A10 were not mapped to regions of previous QTLs, this was consistent with our expectation that it would be a novel locus for VW resistance in this study.

### Identification of candidate genes

Trait–SNP association analysis showed that the peaks associated with VW resistance on A10 were continuous and common in three environments. We infer that it is a major genetic locus responsible for VW resistance in cotton. Comparative genomic analyses have indicated that plant genomes can encode several hundreds of NB‐LRR genes (He *et al*., 2004), and they often occur in clusters at specific loci following gene duplication and amplification events (Chen *et al*., 2015a; Richly *et al*., 2002). Furthermore, many NB‐LRR genes were showed to be colocalized with resistance loci based on the physical or genetic mapping (Daniela *et al*., 2013). In this study, the resistance locus identified in A10 was present to be rich in NB‐LRR genes which occurred in RGA clusters, and there were 16 RGAs in the region of 99‐100 Mb on A10 (Figure S2).

Many resistant genes (R) have been cloned from diverse plant species, most of which encode NB‐LRR proteins (Collier and Moffett, 2009; Wang *et al*., 2015). Effector‐triggered activation of R proteins leads to an array of protective responses, often culminating in a hypersensitive response to prevent further ingress of the pathogen (Greenberg and Yao, 2004; Slootweg *et al*., 2013). NB‐LRR proteins recognize pathogen effectors either directly by physical binding of effectors to R proteins or indirectly through the perception of effector‐induced modifications of host proteins known as guardees, decoys or more generally cofactors by R proteins (Collier and Moffett, 2009; Dodds and Rathjen, 2010). Two NB‐LRR proteins from rice, *RGA4* and *RGA5*, were found to be required for the recognition of the *Magnaporthe oryzae* effectors AVR1‐CO39 and AVR‐Pia by direct binding (Cesari *et al*., 2013). Research in *Arabidopsis* has shown that the TIR‐NBS‐LRR resistance protein RPP1 associated by its LRR domain with the effector ATR1 in *Peronospora p*arasitica, while the Toll/interleukin‐1 receptor (TIR) domain facilitates the induction of the hypersensitive cell death response (Krasileva *et al*., 2010). In this study, the candidate gene CG02, its homologues in *Arabidopsis* encoding NB‐LRR proteins, was shown to be involved in VW resistance response in cotton. However, the molecular mechanisms of effector recognition with the activation of downstream resistance signalling pathways remain to be elucidated.

## Experimental procedures

### Plant materials and phenotypic evaluation

A total of 299 cotton accessions were collected from major breeding institutes across China and the germplasm gene bank of CRI‐CAAS. The population consisted of 263 cultivars or accessions developed from major cotton regions in China (YR, YZR, NW and LN) and 26 introduced from abroad (Foreign group, or FG).

All 299 accessions were planted in a field screening nursery at Shihezi, Xinjiang, China, in 2015 and 2016. The field screening nursery was prepared by consecutively growing susceptible cultivars to build up the inoculum, and supplemented by the distribution of crop residues from heavily infected cotton plants over many years, ensuring that the susceptible control (JM11) would reach an anticipated severity of infection. The field experiments followed a randomized complete block design with three replicates. Each accession was grown in a plot with 30–40 plants in each of two rows, with a distance of 10 cm between plants within each row and 45 cm between rows. The trial management followed standard breeding field protocols. A scale of 0–4 was used to rate disease severity based on the percentage of diseased leaves, where 0 = no symptoms, 1 = less than 25% symptomatic, 2 = 25%–50% symptomatic, 3 = 50%–75% symptomatic and 4 = more than 75% of leaves showing symptoms. These ratings were then converted to a disease index (DI) to estimate the severity of infection for a certain line. The DI was calculated as follows: $\text{DI}=\frac{\sum (d_{c}\times n_{c})}{n_{t}\times 4}\times 100$, where *d*_*c*_ was the disease severity rating, *n*_*c*_ the number of plants with the corresponding disease severity rating and *n*_*t*_ the total number of plants assessed. The DI was further adjusted to create a relative disease index (RDI) to decrease error by a correction factor *K*, defined as: $K=\frac{50.00}{{\text{DI}}_{\mathrm{ck}}}$,where 50.00 was regarded as the standard DI of the susceptible control and DI_ck_ was the actual DI of the susceptible control. RDI was defined as: RDI = DI × K, where DI was the actual DI of the testing lines. When the DI of the susceptible control reached approximately 50, implying uniform and severe infection, disease severity ratings of each cotton line were recorded and DI and RDI were calculated, serving as an evaluation of disease resistance for the cotton line at adult‐plant stage (Zhao *et al*., 2014).

Another phenotypic measure of disease severity was obtained in a controlled glasshouse under a 16/8‐h light–dark photoperiod at 25 °C. The 299 accessions were planted in paper pots using a completely randomized block design with three replicates. Cotton seeds were planted in paper pots (7.5 cm in diameter and 8.5 cm in height) with five seeds per pot and six pots per accession in each replication. The highly virulent *V. dahliae* strain Vd991 was cultured on liquid complete medium at 25 °C for 10 day, after which the concentration of conidia was adjusted to 5 × 10^6^ conidia/mL. Symptom development was recorded from 22 to 25 day and categorized into five grades: 0 = healthy plant, no symptoms on leaves; 1 = one or two cotyledons showing symptoms but no symptoms on true leaves; 2 = both cotyledons and one true leaf showing symptoms; 3 = both cotyledons and two true leaves showing symptoms; and 4 = all leaves showing symptoms, symptomatic leaves dropped, apical meristem necrotic or plant death (Zhao *et al*., 2014). The DI and RDI were calculated according to the same method used in the field screening nursery.

### SNP genotyping and quality control

For each cotton accession, genomic DNA was isolated from fresh leaves of a single plant per accession using CTAB method (Paterson *et al*., 1993). SNP genotyping of the association panel was performed using a SLAF‐seq approach (Sun *et al*., 2013). Sequencing libraries of each accession were constructed through restriction enzymes *Hae*III and *Hpy*166II (New England Biolabs, NEB, USA) that digest cotton genomic DNA into DNA fragments of 364–464 bp. These were selected as SLAFs and prepared for pair‐end sequencing on an Illumina High‐seq 2500 sequencing platform (Illumina, Inc.) at Biomarker Technologies Corporation (Beijing, China). The 125‐bp sequence read at both ends of the fragment in each library was generated using an Illumina Genome Analyzer II (Illumina Inc.) with a barcode approach, to identify each sample. The raw reads (125 bp) were filtered and trimmed according to predefined criteria, and then a 100‐bp paired length sequence was retained at each end. The BWA software was used to map raw paired‐end reads onto the reference genome (*G. hirsutum* v. 1.0) (Zhang *et al*., 2015b). The SLAF groups were generated by reads mapped to the same position. If an accession is only partly digested by restriction enzymes, some reads mapped to the reference genome should overlap two SLAF tags; such reads were assigned to both SLAF tags in that accession. A total of 649,625 high‐quality SLAF tags were thus obtained from each of the 299 genotypes. The GATK (McKenna *et al*., 2010) and SAMtools (Li *et al*., 2009) packages were used to perform SNP calling. All SNPs called by GATK and SAMtools were designated high‐quality; we filtered SNPs with missing rate ≤50% and MAF above 0.05. In total, 85 630 SNPs were identified and used for further analysis.

### LD and population structure analysis

LD was calculated as the squared correlation coefficient (*r* ^2^) of alleles using Haploview 4.2 (Barrett *et al*., 2005). Parameters in the program included MAF (≥0.05) and the missing rate of each SNP (≤50%). A total of 85 630 SNPs distributed evenly across the entire genome were selected for genetic relatedness analyses. PCA based on the same SNP set was carried out using the EIGENSOFT software (Price *et al*., 2006). The phylogenetic tree of the 299 accessions was constructed using MEGA 5.1 program with neighbour‐joining methods (1000 bootstraps) (Tamura *et al*., 2011). The relative kinship matrix of 299 cotton lines was computed using SPAGeDi software (Hardy and Vekemans, 2002).

### Genome‐wide association analysis and haplotype block construction

A total of 85 630 SNPs were used for GWAS with a compressed mixed linear model (CMLM) implemented in the GAPIT software (Lipka *et al*., 2012). The first three PCA values were used as fixed effects in the model to correct for stratification (Price *et al*., 2006). The GWAS threshold was set to *P *<* *1.17 × 10^–5^ (1/total SNPs used, –log_10_ *P* = 4.93). Those with *P*‐values lower than 1.17 × 10^–5^ were defined as significant trait‐associated SNPs; genes that were located within the region 300 kb upstream or downstream of trait‐associated SNPs were identified as candidate associated genes.

Haplotype blocks were constructed using the confidence interval method (Gabriel *et al*., 2002) with Haploview software (Barrett *et al*., 2005), where the Hardy–Weinberg *P*‐value cut‐off was 0.001 and MAF was 0.05.

### RNA isolation and qRT‐PCR analysis

Total RNA was extracted from cotton seedling roots using a Plant RNA Purification Kit (Tiangen, Beijing, China). cDNA was prepared using M‐MLV Reverse Transcriptase and qRT‐PCR analyses were conducted using the SYBR Premix Ex Taq kit (Takara) on a QuantStudio 6 Flex Real‐Time PCR System (Applied Biosystems, Foster City, CA). The primers used for PCR amplification are listed in Table S7 and designed to avoid conserved regions. The cotton 18S gene was used as an internal control to normalize the variance among samples. PCR conditions consisted of an initial denaturation step at 95 °C for 10 min, followed by 40 cycles of denaturation at 95 °C for 15 s, annealing at 60 °C for 30 s and extension at 72 °C for 20 s. Relative expression levels were evaluated using the 2^−ΔΔCT^ method (Livak and Schmittgen, 2001).

### VIGS analysis and detection of VW resistance

For the VIGS assays, five fragments amplified from R genotype (ZZM2) cDNA were integrated into the vector pTRV2 to construct pTRV2:CG02/CG03/CG12/CG13/CG19 and introduced into *Agrobacterium tumefaciens* GV3101. The primers used for the construction of the VIGS vectors are listed in Table S7. Agrobacterium strains harbouring pTRV2: CG02/CG03/CG12/CG13/CG19 plasmid combined with strains harbouring pTRV1 vector were mixed in a 1:1 ratio and co‐infiltrated into the cotyledons of 2‐week‐old cotton plants (ZZM2) with JM11 serving as a susceptible control. The control vector pTRV2:CLA1 was used to evaluate the effectiveness of VIGS. Two weeks later, white leaves were observed in plants in which the CLA1 gene had been targeted by VIGS, and all of plantlets were subjected to *V. dahliae* inoculation with 200 mL of conidial suspension (5 × 10^6^ conidia/mL). VW symptoms were investigated 3 weeks postinoculation. Seedling shoots were cut to investigate vascular wilt under a microscope postinoculation (Chen and Dai, 2010; Liu *et al*., 2013). For in planta fungal biomass quantification, stems of three inoculated plants were harvested at 20 day postinoculation. qRT‐PCR on genomic DNA isolated from the samples was conducted using the SYBR Premix Ex Taq kit (Takara). Verticillium elongation factor 1‐α was used to quantify fungal colonization (Santhanam *et al*., 2013).

## Supporting information


**Figure S1** Overlapping among the SNP loci identified through our genome‐wide association study (GWAS) and quantitative trait loci (QTLs) reported in previous studies.
**Figure S2** Resistance gene analogs (RGAs) in the vicinity of the significant SNP loci identified on A10.
**Figure S3** VIGS analysis of five candidate genes for *V. dahliae* resistance.
**Figure S4** Plant phenotypes for *V. dahliae* resistance as determined by VIGS at 21 day post‐inoculation with *V. dahliae*.

**Table S1** Information of 299 upland cotton accessions.
**Table S2** Analysis of variance (ANOVA) results of VW resistance.
**Table S3** SNP distribution on each chromosome.
**Table S4** Five SNPs on A10 identified in all three environments.
**Table S5** Relative expression levels of candidate genes in qRT‐PCR.
**Table S6** The QTLs of VW resistance from 11 reports of QTL mapping.
**Table S7** Primers used in this study.
