# Supplementary material for: Genome‐wide association study discovered candidate genes of Verticillium wilt resistance in upland cotton (Gossypium hirsutum L.)
Source: Plant Biotechnol J. 2017 Jul 8;15(12):1520–32. doi: 10.1111/pbi.12734 (PMC5698051; doi:10.1111/pbi.12734)
Supplement: Supplementary file 1 — Figure S1 Overlapping among the SNP loci identified through our genome‐wide association study (GWAS) and quantitative trait loci (QTLs) reported in previous studies. Figure S2 Resistance gene analogs (RGAs) in the vicinity of the significant SNP loci identified on A10. Figure S3 VIGS analysis of five candidate genes for V. dahliae resistance. Figure S4 Plant phenotypes for V. dahliae resistance as determined by VIGS at 21 day post‐inoculation with V. dahliae. [file PBI-15-1520-s001.docx]

**Supporting Information**


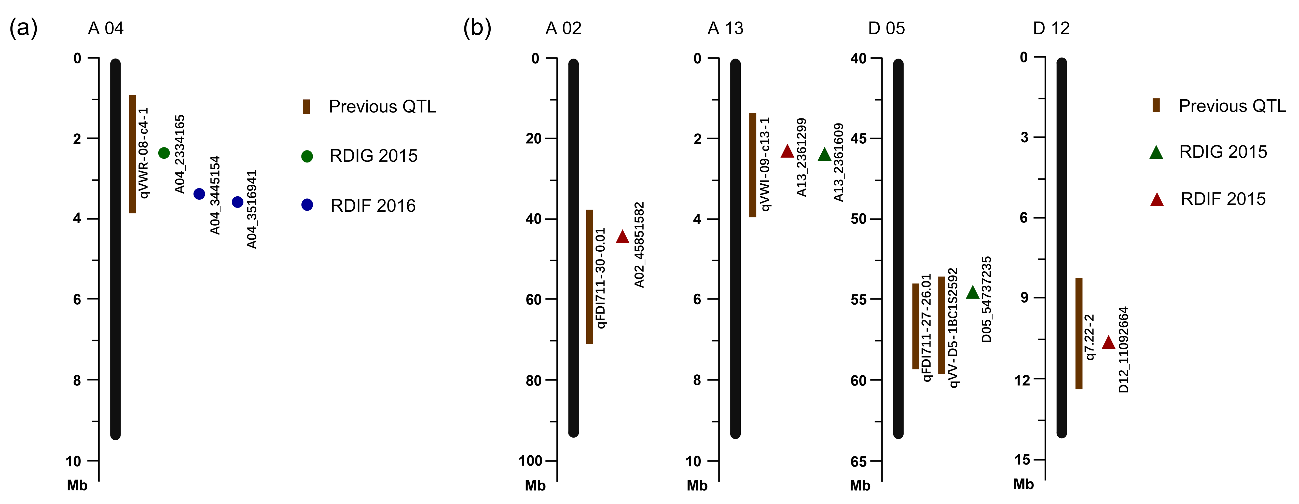


**Figure S1** Overlapping among the SNP loci identified through our genome-wide association study (GWAS) and quantitative trait loci (QTLs) reported in previous studies. (a) Overlapping among the significant SNPs and QTLs. The unit of physical distance for the chromosomes is Mb; brown rectangles represent QTLs from previous studies in the vicinity of the associated SNP loci identified through GWAS; green dot represents the significant SNP identified in RDIG2015; blue dot represents the significant SNP identified in RDIF2016; (b) Overlapping among the peak SNPs and QTLs. The unit of physical distance for the chromosomes is Mb; brown rectangles represent QTLs in the vicinity of the associated SNP loci identified through GWAS; green triangle represents the peak SNP identified in RDIG2015; red triangle represents the peak SNP identified in RDIF2015.


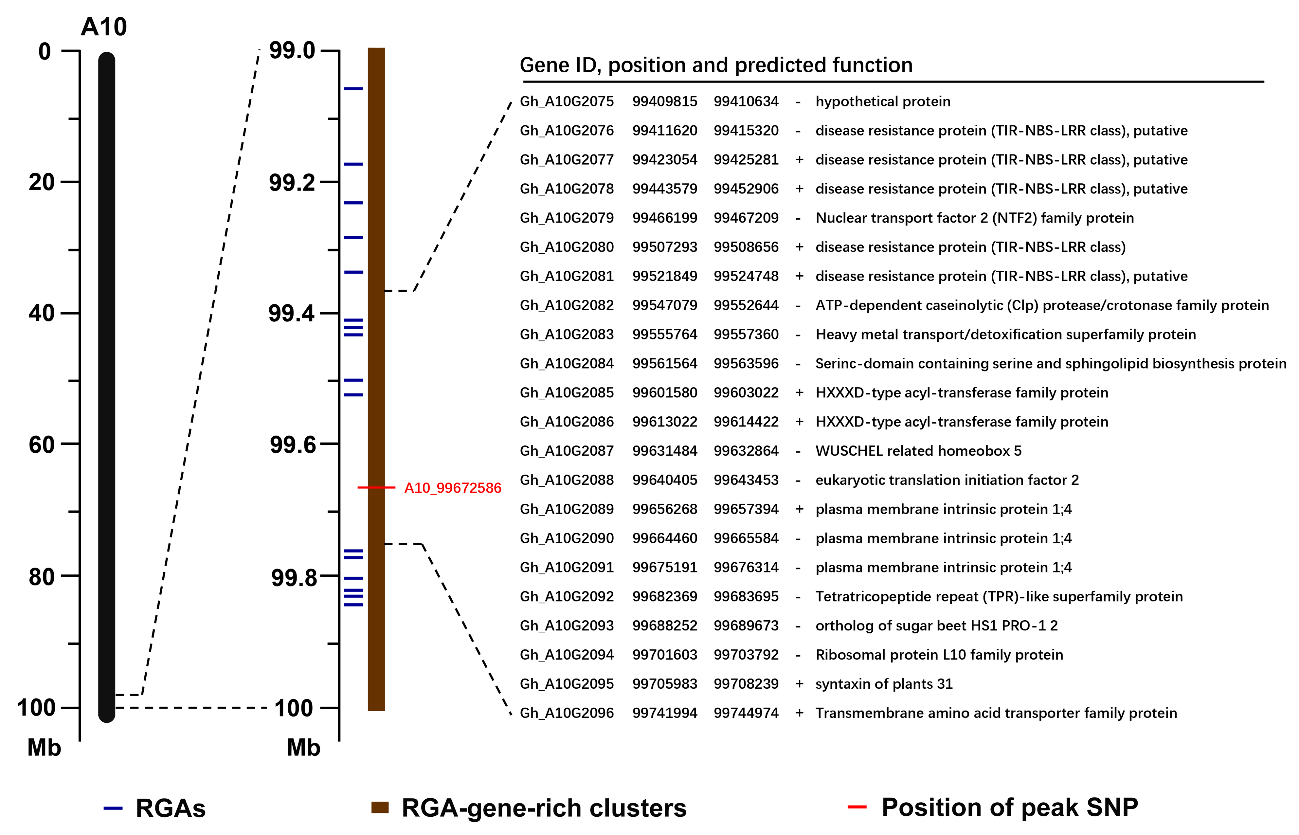


**Figure S2** Resistance gene analogs (RGAs) in the vicinity of the significant SNP loci identified on A10.


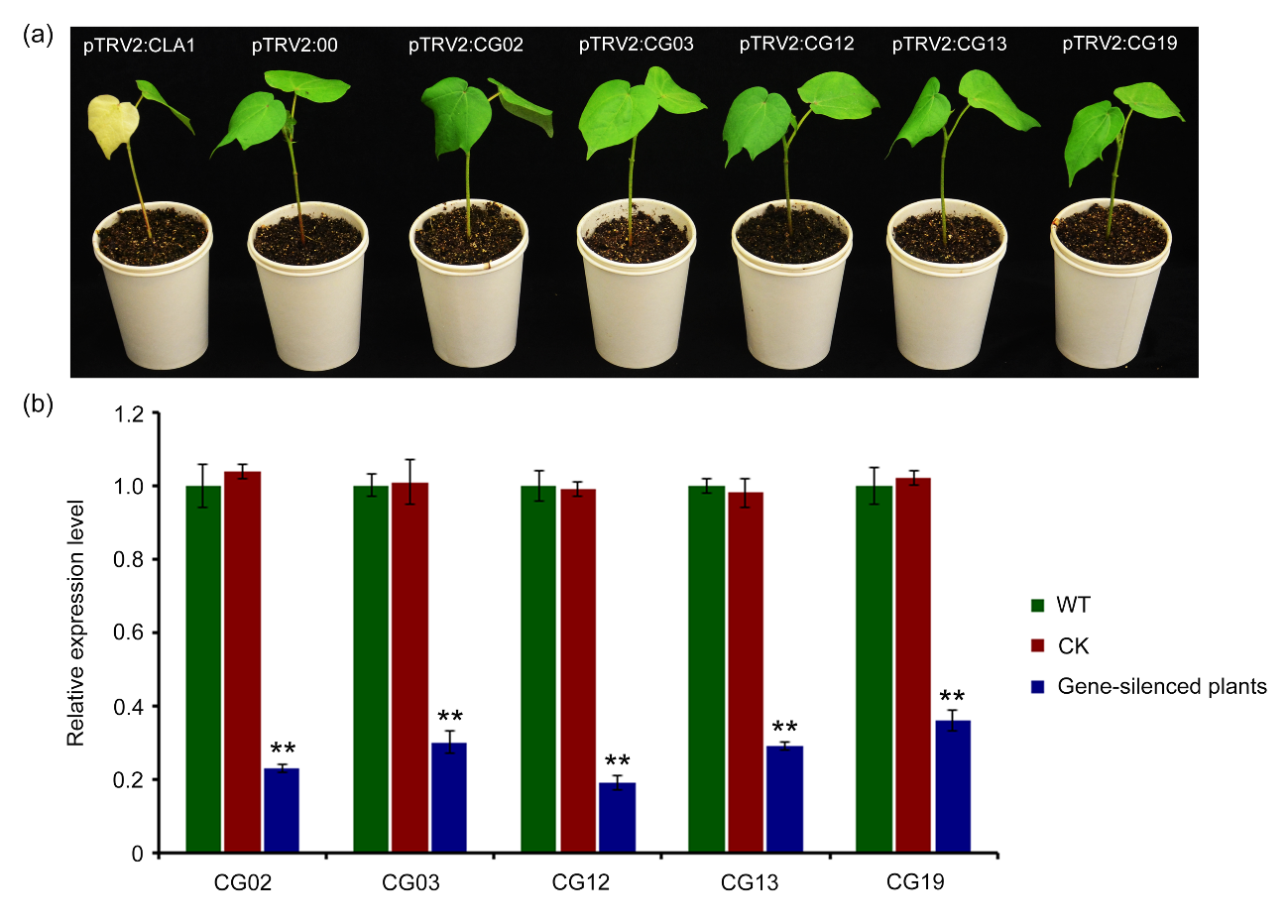


**Figure S3** VIGS analysis of five candidate genes for *V. dahliae* resistance. (a) Phenotypes after VIGS silenced of CLA1 and five candidate genes; (b) Gene expression of CG02, CG03, CG12, CG13, and CG19 in control and silenced plants determined by qRT-PCR analysis. WT and CK represent Zhongzhimian2 (ZZM2) and pTRV2:00 respectively. Error bars were calculated based on three biological replicates using standard deviation. ** indicates significant difference (*P* < 0.01).


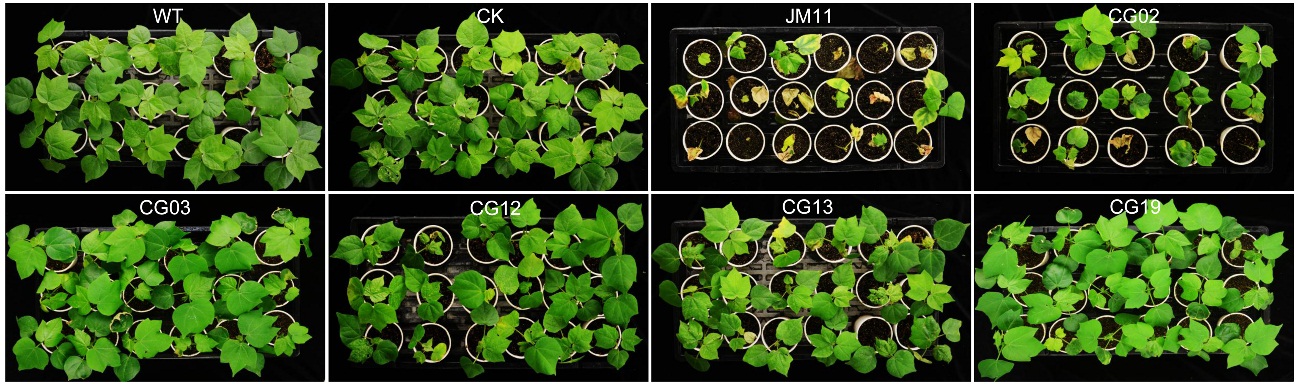


**Figure S4** Plant phenotypes for *V. dahliae* resistance as determined by VIGS at 21 d post-inoculation with *V. dahliae*. WT and CK represent Zhongzhimian2 (ZZM2) and pTRV2:00 respectively. CG02, CG03, CG12, CG13, and CG19 represent gene-silenced plants.
